# Homœopathy—An Introductory Address

**Published:** 1854-02

**Authors:** 


*Homœopathy—An Introductory Address.* By CHARLES A. LEE, M. D.

Prof. Lee has here given the students of Starling Medical College a host of solid facts and irrefragable arguments, which may serve them well, hereafter, in their practice in quack-ridden Ohio. He goes to the fountain-head, and shows a familiarity with Homœopathic doctrines and writings, which evinces his extensive reading. Dr. Lee is well known, both as a thorough scholar, and an honest hater of quackery in every form. He evidently assumes this task of flagellating the Homœopaths *con amore.*

But the grand secret of the success of this empty and superficial doctrine, lies, not in its own strength, but in the weakness of those who receive it. This fact is well stated in the following quotation from near the close of the lecture:

"A person who is ultra in one thing will be ultra in all; a believer in Homœopathy will be, most likely, a believer in spirit-rappings, and mesmerism. Six-sevenths of the followers of Emanuel Swedenborg, it is ascertained, are enthusiastic disciples of Hahnemahnn. A mystic in religion will be a mystic in medicine. Evidence has nothing to do in the making of such converts. *Homœopathicus nascitur non fit.* Here is not faith like a grain of mustard seed that is to remove the mountain, but it is the mountain of faith that is to swallow the mustard seed!"

Introductories, by Wm. M. McPheeters, M. D., of the St. Louis University, and by H. M. Bullitt, M. D., of the Kentucky School of Medicine, have also been received. H.

